# New Synthetic Methods of Novel Nanoporous Polycondensates and Excellent Oxygen Permselectivity of Their Composite Membranes

**DOI:** 10.3390/nano9060859

**Published:** 2019-06-05

**Authors:** Yu Zang, Toshiki Aoki, Masahiro Teraguchi, Takashi Kaneko, Hongge Jia, Liqun Ma, Fengjuan Miao

**Affiliations:** 1 Heilongjiang Province Key Laboratory of Polymeric Composition Material, College of Materials Science and Engineering, Qiqihar University, Wenhua Street 42, Qiqihar 161006, China; jiahongge11@hotmail.com (H.J.); maliqun6166@163.com (L.M.); 2 Faculty of Engineering, Niigata University, Ikarashi 2-8050, Nishi-ku, Niigata 950-2181, Japan; teraguti@eng.niigata-u.ac.jp (M.T.); kanetaka@gs.niigata-u.ac.jp (T.K.); 3 College of Communications and Electronics Engineering, Qiqihar University, Wenhua Street 42, Qiqihar 161006, China; miaofengjuan@163.com

**Keywords:** synthesis, high oxygen permselectivity, nanopore, nanospace, polyphenylacetylene, polycondensates, highly selective photocyclicaromataization (SCAT)

## Abstract

Two kinds of novel nanoporous polycondensates (sc(**Rf**)) have been synthesized by two new preparation methods consisting of polycondensation and highly selective photocyclicaromataization of 1/3 helical cis-cis polyphenylacetylenes with polymerizable groups. By the original methods, new well-defined sheet polymers having nanopores or nanospaces have been synthesized for the first time. Their composite membranes, containing small amounts (1.0 wt%) of sc(**Rf**), had ultrahigh oxygen permeability (*P*o_2_ > 1000 barrer), and their plots were beyond the Robeson’s upper bound line in the graph of oxygen permselectivity (*α* = *P*o_2_/*P*_N2_) versus *P*o_2_. Both *α* and *P*o_2_ values were enhanced by adding only small amounts (1.0 wt%) of sc(**Rf**). One of the sc(**Rf**)s synthesized on the base membrane surface showed the best performance, i.e., *P*o_2_ = 5300 barrer and *α* = 2.5. The membrane surface was effectively covered by sc(**Rf**), judging from the contact angle values. It is thought that nanopores and nanospaces created in and between sc(**Rf**) molecules played an important role for the enhancement of both *α* and *P*o_2_/*P*_N2_.

## 1. Introduction

Permselective membranes have attracted much attention, because they are useful for purifying natural resources and the removal of harmful contaminated component from waste with low energy consumption. Among these, oxygen permselective membranes, which oxygen is able to permeate more selectively than nitrogen, are very important in scientific and practical terms, because separation is difficult and oxygen is a very important substance. The requirements for oxygen permselective membranes are: (1) High oxygen permeability (*P*o_2_), (2) high oxygen permselectivity (*α* = *P*o_2_/*P*_N2_), (3) self-dense membrane-forming ability with no defects and having no ability for separation, and (4) stability [1,2,3,4]. Many materials possessing oxygen-selective adsorption or permeation have been reported, but in general, they have the following problems: (1) there is a trade-off relationship between *P*o_2_ and *α* [5,6,7], and (2) high-ordered compounds showing good selectivities tend to have a low self-dense membrane-forming ability. 

The mechanism of permselectivity is mainly based on the small size difference (molecular-sieving effects) between oxygen (0.34 nm) and nitrogen (0.36 nm). Therefore, their (*α* = *P*o_2_/*P*_N2_) values were not high, and were less than 3.5 when *P*o_2_ values were greater than 1000 barrer. The molecular sieving effects were realized by small nanopores or ultramicropores (<0.7 nm), while ultrahigh permeability (*P*o_2_ > 1000) was caused by large nanopores or micropores (<2 nm) [8,9]. As for membranes with good oxygen permselectivity, two kinds of nanoporous (microporous) polymer membranes are known, that is, polymer intrinsic microporosity (PIM) [9,10,11,12,13,14] and poly(substituted acetylenes) (PA) such as polytrimethylsilylpropyne (PTMSP) [15] and polytrimethylsilyldiphenylacetylene (PDPA) [16,17]. PIMs have high *α* values and show excellent performance. Their plots are on the 2008 upper bound line in the *α*-*P*o_2_ graph; that is, they are the best polymers so far. PAs have extremely high *P*o_2_ and the highest *P*o_2_ of all the polymers. However, since they are linear polymers, their nanopores are formed between the macromolecules. Therefore, their nanopores are very unstable and their good performance is lowered or disappears as a result of aging effects. As more stable nanopores, hypercrosslinked polymers (HCP) [18,19,20], conjugated microporous polymers (CMP) [17,18,19], and covalent organic frame works (COF) [21], the micropores of which have been reported inside their macromolecules as ring structures. However, since they are insoluble, it is impossible to investigate the chemical structures by measuring NMR and GPC and it is also difficult to prepare self-dense membranes with no defects. To solve this problem, mixed-matrix membranes (MMM) [22,23,24,25] have been reported. MMMs consist of organic or inorganic fillers with regular and stable nanopores and base polymers with self-dense membrane-forming ability. However, to obtain better performances in MMMs, it is necessary that the two components create no defects at their interfaces in MMM. To realize this, the compatibility of the two components is important.

**Scheme 1:**
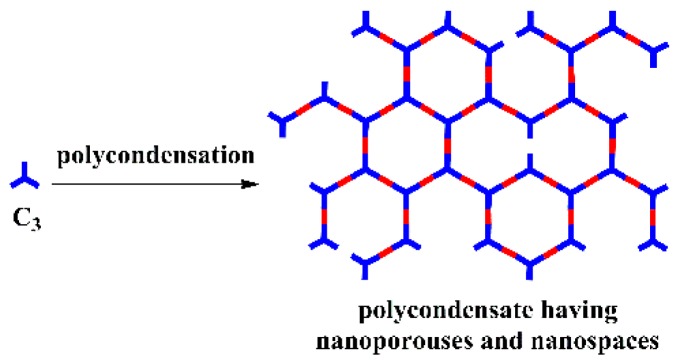
Polycondensation of C_3_ symmetric monomers with three polymerizable groups to polycondensates having nanopores and nonospaces.

The purpose of this study is to synthesize novel soluble polymer sheets having nanopores as fused ring structures or/and nanospaces as hyperbranched structures inside the macromolecules (Scheme 1, right), and then to prepare their MMM consisting of the nanoporous sheet polymer and a base polymer such as PDPA, and finally to realize good performance as oxygen permselective membranes exceeding the upper bound line. In this study, to obtain such nanoporous or/and nanospace-including sheet polymers, we selected polycondensation of C_3_ monomers with three polymerizable groups (Scheme 1). Using this polycondensation reaction, polymer sheets having nanopores as fused ring structures or/and nanospaces as hyperbranched structures inside the macromolecules can be obtained.

**Scheme 2:**
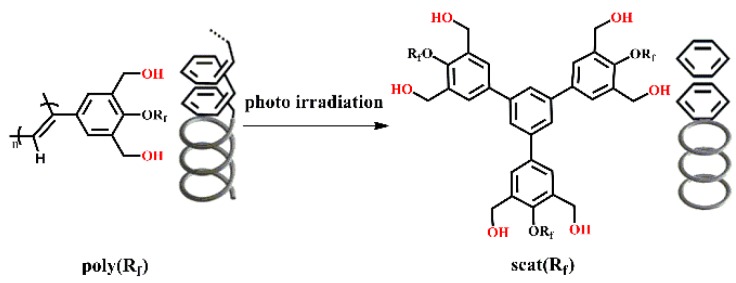
Synthesis of scat(**R_f_**) as C_3_ symmetric monomers (see Scheme 1) by highly selective photocyclic aromatization (SCAT) of 1/3 helical cis-cis polyphenylacetylene (poly(**R_f_**)) having polymerizable groups synthesized by HSSP (see Scheme 4).

**Scheme 3:**
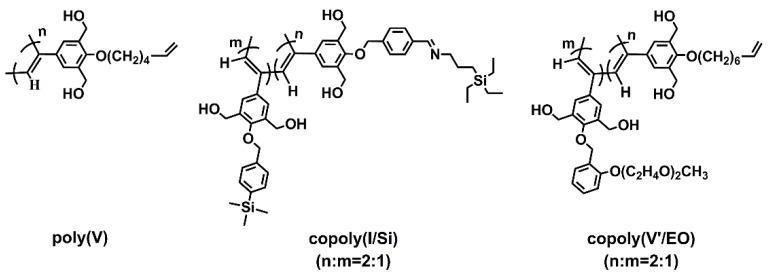
1/3 helical cis-cis (co)polyphenylacetylenes ((co)poly(**R_f_**)) with polymerizable pendant groups as starting compounds for novel nanoporous polycondensates (sc(**R_f_**) = sc(**V**), sc(**I**/**Si**), and sc(**V’**/**EO**), see Scheme 5 and Scheme 6); Poly(**R_f_**) = poly(**V**), copoly(**I**/**Si**), and copoly(**V’**/**EO**). **V**(**V’), I**, **Si**, and **EO** are vinyl-, imine-, silyl-, and ethylene oxide-containing acetylene monomers, respectively.

**Scheme 4:**
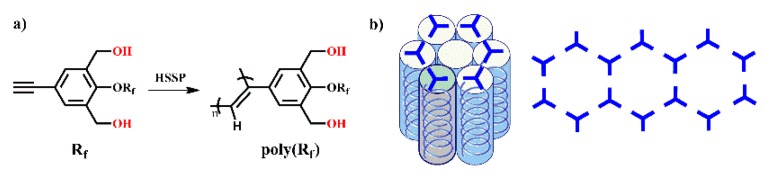
(**a**) Synthesis of 1/3 helical cis-cis polyphenylacetylene (poly(**R_f_**)) as starting compounds of SCAT reaction (Scheme 2) by helix sense-selective polymerization (HSSP) and (**b**) the columnar ordered structure of this polymer in the membrane state as the C_3_ symmetric monomer equivalent templates for the HPS method (Scheme 6).

**Scheme 5:**
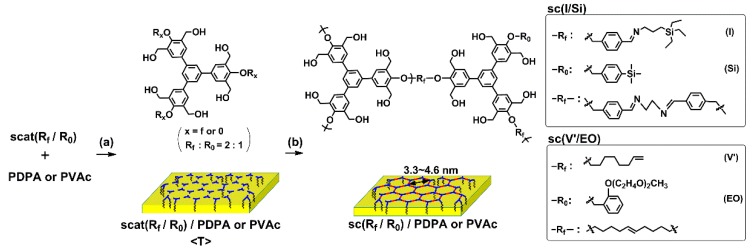
Preparation route to novel nanoporous polycondensate composite membranes (Method 1: HSP method): to polycondensates (sc(**I/Si**) or sc(**V’**/**EO**)) via supramolecular templates (<T>) of scat(**I/Si**) on the membrane surface. (**a**) Fabrication of the blend membranes of scat(**I/Si**) or scat(**V’**/**EO**) with a base polymer (PDPA or PVAc, respectively); (**b**) polycondensation of scat(**I/Si**) with ethylenediamine or polycondensation of scat(**V’**/**EO**) with the Grubbs catalyst on the membrane surface.

**Scheme 6:**
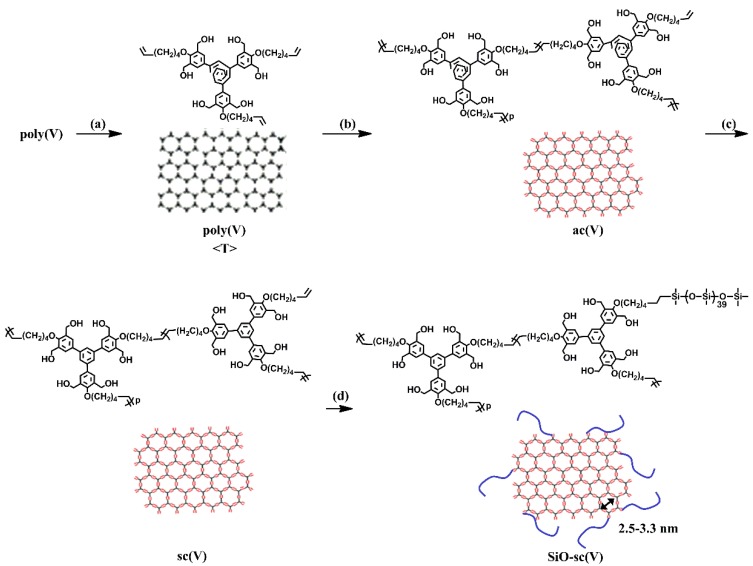
Synthetic route to novel nanoporous polycondensates (Method 2: HPS method): to soluble polycondensates (SiO-sc(**V**)) via supramolecular polymer templates (**<T>**) (Scheme 4b) of pure 1/3 helical cis-cis polyphenylacetylenes with functional groups (polya(**V**)). (**a**) Fabrication of the pure membrane of poly(**V**); (**b**) polycondensation of vinyl pendant groups in poly(**V**) with the Grubbs catalyst to insoluble ac(**V**); (**c**) Highly selective photocyclicaromataization (SCAT) of ac(**V**) to sc(**V**); (**d**) grafting reaction to insoluble sc(**V**) with dimethylsilyl(SiH)-terminated oligosiloxane to soluble SiO-sc(**V**).

To synthesize the C_3_ monomers with three polymerizable groups, we applied our original selective photoreaction called SCAT (Scheme 2) [26,27,28,29]. The SCAT reaction needs our original polymers as the starting compounds, that is, 1/3 helical cis-cis polyphenylacetylenes(poly(**Rf**), Scheme 3) with polymerizable pendant groups, which can be synthesized by helix sense-selective polymerization (Scheme 4) [30,31,32,33,34]. Scat(**Rf**), as the C_3_ monomers with three polymerizable groups, were synthesized by SCAT of poly(**Rf**) only by photoirradiation (Scheme 2). Scat(**Rf**)s were polymerized on the membrane surface to obtain sc(**Rf**)s, that is, nanoporous or nanospace-containing sheet polymers on the membrane surface (HSP method, Scheme 5). The resulting composite membranes were used directly for oxygen permeation. As an alternative method to synthesize similar polymer sheets having nanopores, the following method was developed in this study. Poly(**Rf**) is a tightly helical main chain, and is therefore very rigid. As a result, it is known that the polymer has columnar ordered structures (Scheme 4). In addition, since it is 1/3 helical main chain, it can be regarded as a C_3_ monomer equivalent. Therefore, it can be used as a template polymer for insoluble nanoporous polymers(ac(**Rf**)s) and insoluble nanoporous sheet polymers(sc(**Rf**)s), as shown in Scheme 6 (HPS method). To solubilize sc(**Rf**)s, grafting by oligosiloxane chain was carried out. The blend solution of the solubilized sc(**Rf**)s and base polymers were cast to prepare their composite membranes, which were used for oxygen permeation. Since these two synthetic methods (HSP and HPS methods, Scheme 5 and Scheme 6) are a combination of our two original reactions, that is, helix sense-selective polymerization (HSSP, Scheme 4) and a photoselective solid-state reaction (SCAT, Scheme 2), they are quite new and original. Using these two novel methods, good new permselective composite membranes containing well-defined sheet polymers having nanopores or nanospaces were obtained for the first time.

## 2. Results and Discussion 

### 2.1. Two New Synthetic Routes of Novel Nanoporous Polycondensates (sc(**Rf**)s) and Preparation of Their Composite Membranes Based on Poly(p-trimethylsilyldiphenylacetylene)(PDPA) or Poly(Vinyl Acetate) (PVAc)

To obtain novel nanoporous polycondensates (sc(**Rf**)s = sc(**V**), sc(**I**), sc(**I**/**Si**), and sc(**V’**/**EO**)), poly(**Rf**)s (=poly(**V**), poly(**I**), copoly(**I**/**Si**)(molar ratio = 2:1), and copoly(**V’**/**EO**) (molar ratio = 2:1)) as starting 1/3 helical cis-cis polyphenylacetylenes with polymerizable pendant groups were synthesized by (co)polymerization of the corresponding monomers and comonomers which have two hydroxyl groups and the second polymerizable group such as vinyl (**V**,**V’**) and imine(I) group (Scheme 3).

Novel nanoporous polycondensates (sc(**Rf**)s) were synthesized by the following two new multi-step routes: **Method 1** (**HSP** method, Scheme 5): (i) highly selective photocyclic aromatization (**SCAT**) reaction of 1/3 helical cis-cis copolyphenylacetylenes(coply(**R_f_**/**R_0_**)s = coply(**I**/**Si**) and coply(**V’**/**EO**)) (Scheme 3) having polymerizable pendant groups such as imno(I) or vinyl(V’) groups and also anchoring groups such as silyl(**Si**) or ethylene oxide(**EO**) groups to yield scat(**R_f_**/**R_0_**)s including scat(**R_f_**) as a C_3_ monomer, (ii) polycondensation reaction of the polymerizable groups in scat(**R_f_**/**R_0_**)s including scat(**R_f_**) on the surface of the base membranes, poly(*p*-trimethylsilyldiphenylacetylene)(PDPA) or poly(vinyl acetate) (PVAc), respectively; **Method 2** (**HPS** method, Scheme 6): (i) ADMET polycondensation reaction of vinyl pendant groups of 1/3 helical cis-cis polyphenylacetylenes(poly(**V**)) (Scheme 3) in columnar structures of the membrane state as a C_3_ monomer equivalent (Scheme 4) to produce ac(**V**), and then (ii) highly selective photocyclic aromatization (**SCAT**) reaction of ac(**V**) to yield sc(**V**), followed by (iii) grafting reaction of the insoluble parts of sc(**V**) with oligosiloxanes having a SiH end group to give soluble SiO-sc(**V**).

By the **HSP** method (Scheme 5), i.e., polycondensation of scat(**I**/**Si**) on PDPA and scat(**V’**/**EO**) on PVAc, PDPA membranes whose surfaces were covered by sc(**I**/**Si**), and PVAc membranes whose surfaces were covered by sc(**V’**/**EO**) were obtained, as shown in Table 1 and Table 2. Judging from the contact angle values of water droplets for the PDPA-based composite membranes (Table 1), it was found that the membrane surfaces became more hydrophilic by the presence of scat(**I**/**Si**) and sc(**I**/**Si**). This indicates that the scat(**I**/**Si**) and sc(**I**/**Si**) were effectively accumulated on the PDPA membranes (Scheme 5). In the case of PVAc-based composite membranes (Table 2), we were able to measure the GPC of the solution of the composite membrane of sc(**V’**/**EO**) and PVAc, because they were totally soluble. From the GPC results detected on UV (since PVAc has no absorption for the UV, sc(**V’**/**EO**) and scat(**V’**/**EO**) could be detected) (Figure S1), we confirmed that the polycondensation proceeded on the membrane surface, although the molecular weight was low as shown in Table 3 and Figure S1. In the case of polycondensation of scat(**I**/**Si**) on PDPA, since sc(**I**/**Si**) and PDPA could not be distinguished in GPC detected by UV, the molecular weight could not be estimated. However, since the resulting sc(**I**/**Si**) contained an insoluble part, their molecular weights may not be low, and may be at least as high as that of sc(**V’**/**EO**). The resulting composite membranes were directly used for oxygen permeation measurements as they were.

**Figure 1:**
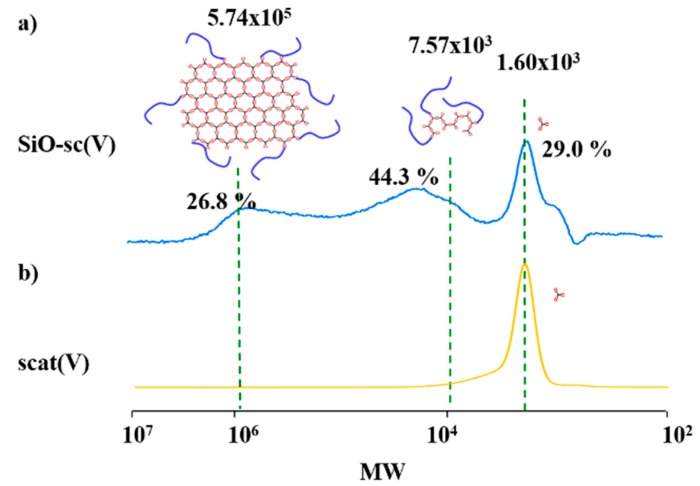
GPC traces of (**a**) novel nanoporous and nanospace-containing polycondensates with oligosiloxane grafts (SiO-sc(**V**)) synthesized by HPS method (Scheme 6) and (**b**) scat(**V**) as reference.

**Figure 2:**
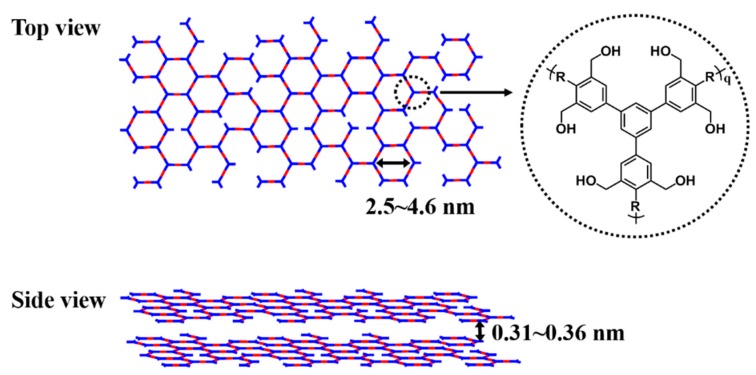
Possible intramolecular and intermolecular nanopores and nanospaces of sc(**V**) and sc(**I**/**Si**).

On the other hand, by the **HPS** method for poly(**V**), followed by grafting reaction with oligosiloxanes having a SiH end group (Scheme 6), soluble SiO-sc(**V**)s containing a very high molecular weight part (=10^5^) were obtained as shown in Figure 1 and Table 3. Although it is the mixture of three components with different molecular weights as shown in Figure 1, it surely contains polycondensates having nanopores or nanospaces inside their molecules, judging from their chemical structures and molecular weights (Figure 2). The possible size of the nanopores or nanospaces are about 2.5–4.5 nm (Figure 2). The possible distance between the sheet polymers in Figure 2 was estimated based on the one for SCAT products we reported previously [26].

**Figure 3:**
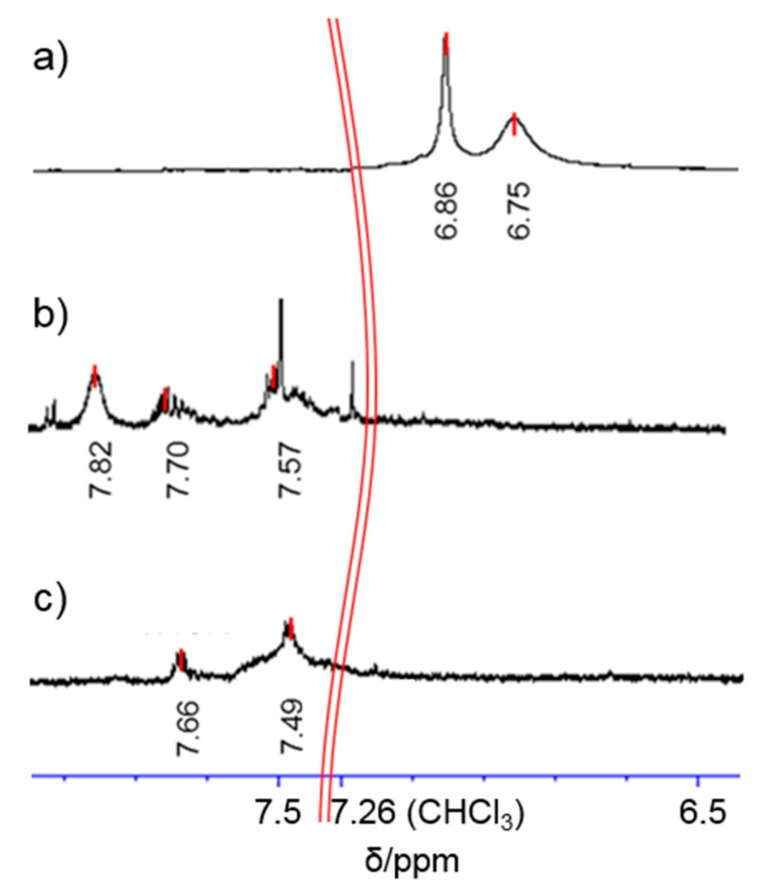
The aromatic region of ^1^H-NMR of novel nanoporous and nanospace-containing polycondensates (SiO-sc(**V**)) synthesized by the HPS method (Scheme 4), together with its related compounds; (**a**) scat(**V**), (**b**) soluble part of sc(**V**), and (**c**) SiO-sc(**V**) synthesized from insoluble part of sc(**V**).

Figure 3 shows the aromatic region of ^1^H-NMR of SiO-sc(**V**) together with its related compounds; (a) scat(**V**), (b) soluble part of sc(**V**) (which was not used for the grafting reaction to SiO-scat(**V**)), and (c) SiO-scat(**V**) synthesized from insoluble sc(**V**). Because the soluble sc(**V**) had low molecular weight, it may not have contained crosslinked structures, fused rings, or sheet structures, and may have contained mainly hyperbranched structures. In fact, its NMR had more than three peaks (Figure 3b). Although the clear assignment for dendritic (D), linear (L) and terminal (T) in the hyperbranched structures is difficult, the peak for the soluble sc(**V**) at the lowest magnetic field (7.8 ppm) may be assigned as L. On the other hand, there are no peaks around 7.8 ppm for L and around 6.8 ppm for T appeared for SiO-sc(**V**) derived from insoluble sc(**V**). This may indicate high content of D. Therefore, SiO-sc(**V**) may contain more fused ring structures (stable nanopores) or more developed hyperbranched structures (stable nanospaces). In conclusion, we think highly developed sheet structures may be formed in SiO-sc(**V**) synthesized by the HPS method.

Composite membranes of the nanoporous polycondensates having the siloxane grafts (SiO-sc(**V**)s) by the **HPS** method and poly(*p*-trimethylsilyldiphenylacetylene) (PDPA) were prepared by solvent cast of the blend solution and they were used for oxygen permeation measurements. Judging from the contact angle (θ) values of the composite membranes (the values for these composite membranes were lower than that for pure PDPA and very close to that for pure scat(**V**) (see Table 3)), the membrane surface was effectively modified by SiO-sc(**V**)s (Table 3).

In summary, when the two methods (HSP and HPS methods) are compared, in the case of the HSP method, sc(**Rf**)s were synthesized by polycondensation of the SCAT products (scat(**I**/**Si**) and scat(**V’**/**EO**)) as a template precursor supramolecularly assembled on the membrane surface. Therefore, more effective surface accumulation of scat(**I**/**Si**) was realized. Instead, the molecular weights were lower than that of sc(**V**) by HPS method, because scat(**I**/**Si**) contains **R_0_** (**=Si**) units, which have no polymerizable group but work as an anchoring group, and therefore the functionality of the SCAT products was lower than three. On the other hand, in the case of the HPS method, insoluble ac(**V**)s and sc(**V**)s were synthesized first, and then the insoluble sc(**V**)s, which may have higher molecular weights, were converted to soluble siloxane-grafted sc(**V**)s. In addition, the composite membranes were prepared from the siloxane-grafted sc(**V**). Therefore, the molecular weights of sc(**V**)s by the HPS method were much higher than those of sc(**I**/**Si**)s by the HSP method, but surface accumulation of the sc(**V**)s by HPS method was less effective than that in the HSP method. This is supported by the contact angle values in Table 1 and Table 3.

As a reference, we synthesized soluble and low molecular weight sc(**V**) having no sheet and nanoporous structures by polycondensation of scat(**V**) in its pure membrane states (Table 4 and Scheme S1). In this condition, since scat(**V**) was self-assembled and formed a 1D structure by π-π stacking (see Figure S2), the resulting sc(**V**) must not be sheet shaped and will have no large nanopores (around 2.5–4.5 nm), only having small nanopores or nanospaces around 0.31–0.36 nm based on the π-π stacking (Figure 2 and Figure S2). In addition, in contrast to the higher molecular weight sc(**V**)s prepared by the HPS and HSP methods described above, it was soluble because of its low molecular weight. In addition, therefore, the composite membranes based on PDPA were prepared by solvent cast of the blend solution.

### 2.2. Excellent Oxygen Permselectivity of the Novel Nanoporous Polycondensate (sc(**Rf**)) Composite Membranes by the New Preparation Methods

**Figure 4:**
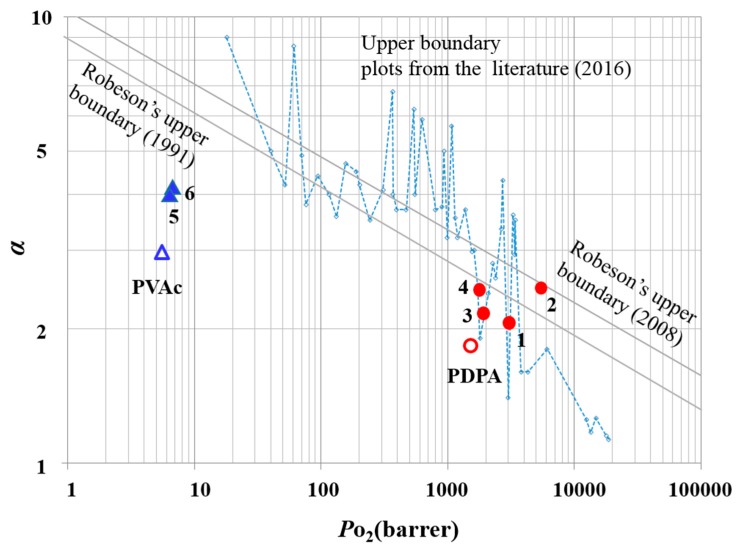
Oxygen permselectivity of the two kinds of composite membranes of novel nanoporous polycondensates (SiO-sc(**V**), sc(**I/Si**) or sc(**V’**/**EO**)) based on ●: poly(*p*-trimethylsilyldiphenylacetylene)(PDPA) or ▲: poly(vinyl acetate) (PVAc) (○: pure PDPA) or ∆: pure PVAc) with upper boundary lines.

**Figure 5:**
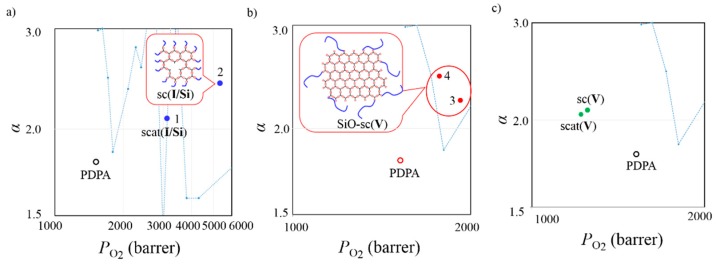
Oxygen permselectivity of the two kinds of composite membranes of novel nanoporous polycondensates (SiO-sc(**V**), sc(**I/Si**) or sc(**V’**/**EO**)) based on PDPA together with composite PDPA membranes of sc(**V**) having no nanopores and nanospaces as reference. (**a**) For SiO-sc(**V**)-containing PDPA membranes prepared according to method 1 (HSP method) (Scheme 5), insert: schematic figure of sc(**I/Si**); and (**b**) for sc(**I/Si**)-containing PDPA membranes prepared according to method 2 (HPS methods) (Scheme 6), insert: schematic figure of SiO-sc(**V**); (**c**) for sc(**V**)-containing PDPA membranes prepared according to method 3 (see Scheme S1); ------: upper boundary plots from the literature (2016).

Both oxygen permselectivities (*α*) and permeabilities (*P*o_2_) for all the composite membranes of novel nanoporous polycondensates (sc(**Rf**) = SiO-sc(**V**), sc(**I/Si**) or sc(**V’**/**EO**)) based on poly(*p*-trimethylsilyldiphenylacetylene) (PDPA) or poly(vinyl acetate) (PVAc) were higher than those for the base membranes, PDPA or PVAc, respectively. In other words, by adding small amounts (1.0 wt%) of the nanoporous polycondensates, both *P*o_2_ and *α* were effectively and simultaneously improved. They were higher than those for pure PDPA and PVAc membranes (Figure 4). In addition, the composite membranes based on PDPA showed very good performances (Figure 4 and Figure 5a,b, Nos. 1–4). Their plots in the *α*-*P*o_2_ graph are very close to or beyond the 1991 upper bound lines. In addition, their *P*o_2_ values were ultrahigh. For gas separation membranes, high permeability is the most important thing. In addition, in this highly permeable region there were no actual plots close to or above the upper bound line, as shown by the upper boundary plots (the dotted line). These plots are actually beyond the actual upper boundary plots (the dotted line). When we compared the two composite membranes prepared by the two methods, that is the HSP and HPS methods (Figure 5a,b, respectively), the former (Nos. 1 and 2 in Figure 4) was a little better than the latter (Nos. 3 and 4 in Figure 4), although the molecular weights of the polycondensates in the latter were higher than those in the former. As a reference, in the composite membrane of low molecular weight soluble sc(**V**) having no large nanopores as shown in Figure 5c, *α* increased but *P*o_2_ decreased. This is different from the two nanoporous sheets sc(**V**) (Figure 5a,b).

### 2.3. The Reason for the Excellent Oxygen Permselectivity of the Novel Nanoporous Polycondensate Composite Membranes by the New Preparation Methods

Contact angle values decreased and were close to values for pure sc(**Rf**) membranes (which were low in strength, but their contact angles could be measured) by adding only small amount (0.5–1.0 wt%) of sc(**Rf**)s having hydroxyl groups, and they were lower compared with the base hydrophobic membranes in all the PDPA composite membranes (Table 1 and Table 3). Therefore, sc(**Rf**)s were accumulated in the surface of PDPA and covered the surface effectively. In other words, very thin or nanomembranes of sc(**Rf**)s may have formed on the surface. Therefore, *α* increased without any drop in *P*o_2._

With respect to the reason for the enhancement of *α* by sc(**Rf**)s, there are two possibilities: one is micropores (large nanopores) of 2.5–4.5 nm (see Figure 2, Scheme 5 and Scheme 6) inside the sc(**Rf**)s molecules, and the other is molecular spaces (small nanospaces) of 0.31–0.36 nm (ultramicropores) between two planar π-π stacking molecules of sc(**Rf**)s and scat(**Rf**)s (Figure 2). Judging from the molecular sizes of oxygen and nitrogen (0.36 and 0.38 nm, respectively), the latter (ultramicropores) may be the main reason for the enhancement of *α* based on the molecular sieving effect, although the former (microspores) can also contribute to the *α* enhancement because they can reduce their pore sizes by tilted stacking.

Surprisingly, in addition to the *α* enhancement, *P*o_2_ also increased in the case of nanoporous sc(**Rf**)s having 2.5–4.5 nm nanopores or nanospaces, as described above. Since in the case of the composite membrane of low molecular weight soluble sc(**V**) having only ultramicropores (small nanospaces) but no micropores (large nanopores) described above (Figure 5c), *α* increased but *P*o_2_ decreased. Therefore, the micropores (nanopores) were effective for retaining and enhancing *P*o_2_. A similar conclusion has been described in some papers [8,9].

As described above, the nanoporous sc(**Rf**)s composite membranes prepared by HSP methods on the membrane surface (Scheme 5) showed better performances (Figure 4 and Figure 5) than those by HPS methods followed by grafting reaction (Scheme 6), although the molecular weight of the nanoporous sc(**Rf**)s by HSP methods must be much lower. This may be because the polycondensates in the former membranes covered the membrane surface more effectively than those in the latter. This speculation is supported by the contact angle values (Table 1 and Table 3).

## 3. Experimental Procedure

### 3.1. Materials

All the solvents used for reactions were distilled as usual. The polymerization initiator, Hoveyda-Grubbs catalyst 2nd generation, purchased from Aldrich Chemical Co., Inc. (Saint Louis, MO, USA), was used as received. The silicon-containing reagents were purchased from Shinetsu Chemical Co., Ltd. (Tokyo, Japan), and used as received. All the monomers and (co)polymers (Scheme 3) were synthesized according to our previous reports [35,36]. Dimethylsilyl(SiH)-terminated oligosiloxane was synthesized according to our previous report [37]. Poly(1-phenyl-2-*p*-(trimethylsilyl)phenylacetylene) (PDPA) was synthesized according to the literature [38].

### 3.2. Preparation of sc(**I**/**Si**)/PDPA and sc(**V’**/**EO**)/PVAc Composite Membranes (Method 1, HSP Method, Scheme 5)

#### 3.2.1. Synthesis of scat(R_f_/R_0_) by SCAT of Copoly(R_f_/R_0_) (Scheme 4) in the Membrane State 

Copoly(**I**/**Si**) (monomer molar ratio = 2:1) was irradiated by visible light ((400–500 nm, 23,000 lx) in the membrane state under nitrogen for 7 days to give scat(**I**/**Si**). Visible light (400–500 nm) irradiation was carried out by using a 300 W of Xe lamp (Asahi Spectra, MAX-302 with vis mirror module, Torrance, CA, USA) through a cutoff filter (Asahi Spectra, LUX400 (λ > 400 nm), XF541 (λ < 510 nm), and XF546 (λ < 610 nm). Similarly, scat(**V’/EO**) was synthesized from copoly(**V’/EO**) (n:m = 2:1) in the membrane state. [26]

#### 3.2.2. Preparation of scat(I/Si)/PDPA and scat(**V’**/**EO**)/PVAc Composite Membrane as a Template<T> (Scheme 5a)

A toluene solution (3.00 mL) of the base polymer PDPA (49.5 mg) and a THF solution (0.100 mL) of scat(**I**/**Si**) (0.500 mg, 1.0 wt%) were blended. In addition, then the blend solution was cast on a poly(tetrafluoroethylene) sheet (100 cm^2^). After the solvent was evaporated for 24 h at room temperature, the membrane was detached from the sheet and dried in vacuo for 24 h at room temperature. In the case of scat(**V’**/**EO**)/PVAc blend membranes, the casting solvent was THF.

#### 3.2.3. Synthesis of sc(I/Si) by Polycondensation of scat(I/Si) on PDPA Membrane Surface (Scheme 5b)

A scat(**I**/**Si**)/PDPA membrane was immersed into ethylenediamine for 24 h. After the reaction, the membrane was dried in *vacuo* for 24 h to give sc(**I**/**Si**)/PDPA blend membrane. The membrane was directly used for oxygen permeation measurement.

#### 3.2.4. Preparation of sc(**V’**/**EO**)/PVAc Blend Membrane (Scheme 5b)

A scat(**V’**/**EO**)/PVAc blend membrane was immersed into a solution of Hoveyda-Grubbs catalyst 2nd generation (1.00 mM) in hexane for 24 h. After the reaction, the membrane was dried in vacuo for 24 h to give sc(**V’**/**EO**)/PVAc blend membrane. The membrane was directly used for oxygen permeation measurement. The blend THF solution was used for GPC measurement.

### 3.3. Synthesis of SiO-sc(V) and Preparation of SiO-sc(V)/PDPA Composite Membranes (Method 2, HPS Method, Scheme 6)

#### 3.3.1. Preparation of Poly(V) Membrane as a Template<T> (Scheme 6a)

A solution of poly(**V**) in tetrahydrofuran (THF) (2~3 wt%) was cast on a poly(tetrafluoroethylene) sheet (100 cm^2^), and the solvent was evaporated for 24 h at room temperature and dried in vacuo. After immersing in methanol, the self-supporting membrane was detached from the sheet, and dried in vacuo to give the poly(**V**) membrane.

#### 3.3.2. Synthesis of Insoluble ac(V) by ADMET Polymerization of Pendant Vinyl Groups in Poly(V) in the Membrane State (Scheme 6b)

A poly(**V**) membrane (20.6 mg) was immersed into ethyl acetate solution of Hoveyda-Grubbs catalyst 2nd generation (1.00 mM) for 24 h. After the reaction, the membrane was immersed in methanol (50.0 mL) for 24 h. In addition, then the resulting membrane was dried in vacuo for 24 h to give ac(**V**) as an orange membrane with yield of 93.5%. The conversion of metathesis was 51.4% by IR. IR(cm^−1^, film): 3600-3100(OH), 3078(CH=CH_2_), 2925, 2854(CH_2_), 1641(C=C), 1464(C-O-C), 864(trisubstituted olefin).

#### 3.3.3. Synthesis of Insoluble sc(V) by SCAT of Insoluble ac(V) in the Membrane State (Scheme 6c)

An ac(**V**) membrane was irradiated under nitrogen by visible light ((400–500 nm, 23,000 lx) for 7 days. After the completion of SCAT reaction, the membrane was immersed into THF. After the resulting insoluble part in THF was dried in vacuo for 24 h, the sc(**V**) membrane was obtained in 81.6% yield.

#### 3.3.4. Synthesis of Soluble SiO-sc(V) by Grafting of Dimethylsilyl(SiH)-Terminated Oligosiloxane to Insoluble sc(V) in the Membrane State (Scheme 6d)

To a two neck flask containing dimethylsilyl(SiH)-terminated oligosiloxane (120 mg, 39.3 μmol), 0.750 mL of THF (3.00 mL) solution of hydrogen hexachloroplatinate(IV) hexahydrate (3.80 mg, 7.10 μmol) was added. In addition, the mixture was then added to another two neck flask containing the sc(**V**) membrane (8.76 mg), and stirred for 48 h at 60 °C. After filtration, the filtrate was washed with ethyl acetate and brine, and then dried with anhydrous MgSO_4_. After filtration and evaporation, the product was dried in vacuo for 24 h to give SiO-sc(**V**) as a brown viscous liquid in 24.3% yield. It was soluble in THF and hexane.

#### 3.3.5. Preparation of SiO-sc(V)/PDPA Composite Membrane

A toluene solution (3.00 mL) of the base polymer PDPA (49.5 mg) and THF solution (0.100 mL) of the SiO-sc(**V**) (0.500 mg, 1.0 wt%) were blended. In addition, then the blend solution was cast on a poly(tetrafluoroethylene) sheet (100 cm^2^). After the solvent was evaporated for 24 h at room temperature, the membrane was detached from the sheet and dried in vacuo for 24 h at room temperature.

### 3.4. Measurements

Average molecular weights (*M*_w_ and *M*_n_) were estimated by gel permeation chromatography (GPC) (tetrahedron as an eluent, polystyrene calibration) using JASCO Liquid Chromatograph instruments with PU 2080, DG 2080 53, CO 2060, UV 2070, and two polystyrene gel columns (Shodex KF 807L). NMR spectra were recorded on a FT-NMR VNMR700NB at 400 MHz or 700 MHz for ^1^H and ^13^C. IR spectra were recorded on a JASCO FTIR 4200 spectrometer. Contact angles of distilled water droplets on the air-side surface of the membranes were measured at 25 °C with a DM301, Kyowa Interface Science Co., Ltd. Oxygen and nitrogen permeability coefficients (*P*_O2_ and *P*_N2_: cm^3^ (STP) cm cm^−2^ s^−1^ cmHg^−1^) and the oxygen separation factor (*α* = *P*_O2_/*P*_N2_) were measured by a gas chromatographic method using YANACO GTR-10 according to Ref. [39,40].

## 4. Conclusions

**Figure 6:**
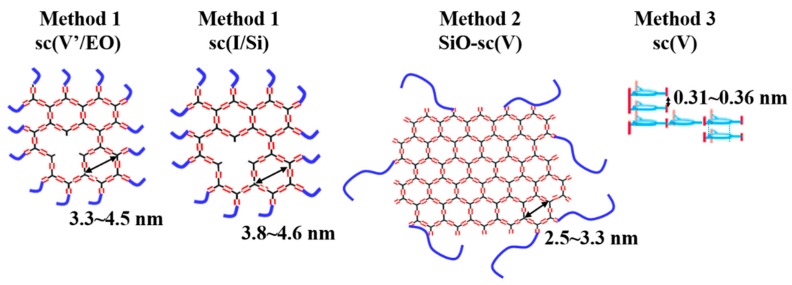
The size of nanopores or nanospaces of sc(**V’**/**EO**) and sc(**I/Si**), SiO-sc(**V**), and sc(**V**) prepared by the three methods, respectively.

Polycondensates (sc(**Rf**)s) having 2.5–4.5 nm nanopores or nanospaces in the macromolecules were synthesized using new multi-step preparation methods consisting of polycondensation of C_3_ monomers such as scat(**V**) or C_3_ monomer equivalent such as poly(**V**) and highly selective photocyclicaromataization(SCAT) of 1/3 helical cis-cis polyphenylacetylenes with polymerizable pendant groups such as vinyl or imino groups. Using these original methods, new well-defined sheet polymers having nanopores or nanospaces were synthesized for the first time. The PDPA-based composite membranes containing small amounts of sc(**V**) or SiO-sc(**V**) on the membrane surface had ultrahigh oxygen permeability (*P*o_2_ > 1000 barrer) and their plots were beyond the Robeson’s 1991 upper bound line in the graph of oxygen permselectivity (*α* = *P*o_2_/*P*_N2_) versus oxygen permeability (*P*o_2_) (Figure 4). By using the new preparation methods, we achieved some of the best performances as oxygen permselective membranes. Both *α* and *P*o_2_ values were enhanced by adding only small amounts (1.0 wt%) of sc(**Rf**)s. In particular, the composite membrane containing sc(**I/Si**) synthesized on the base membrane surface showed the best performance, *P*o_2_ = 5300 barrer and *α* = 2.5, which is very close to the Robeson’s 2008 upper bound line (Figure 4). Since both *α* and *P*o_2_/*P*_N2_ values were enhanced by only adding small amounts (1.0 wt%) of sc(**Rf**)s, their membrane surface was thought to be effectively covered by sc(**Rf**)s. The effective surface modification is supported by the contact angle values. It was thought that nanopores and nanospaces created in and between sc(**Rf**)s play an important role for the simultaneous enhancement of *α* and *P*o_2_. The large nanopores and/or nanospaces (2.5–4.5 nm) in sc(**Rf**)s may be effective for enhancing *P*o_2_ and the small nanospaces (0.31–0.36 nm) between sc(**Rf**)s and scat(**Rf**)s were effective for enhancing *α* (Table 5 and Figure 6).

Although the sizes of the nanopoes and nanospaces in the nanosheets such as sc(V) were estimated by the experimental results for the precursors, judging from the improvements of the permselectivities for oxygen and nitrogen we consider that such micropores surely exist in the membranes. Similar discussion about the effects of micropores and ultramicropores on permselectivities has been described in some reports [8,9,10,11,12,13,14]. By new preparation methods based on our original two reactions (HSSP and SCAT), we have successfully prepared composite membranes containing sheet polymers having well-defined nanopores and nanospaces. Because the method is not limited and can be used for other similar (co)monomers as the starting compounds, we can obtain many kinds of well-defined polymers having different sizes of nanopores by changing the starting monomers and comonomers. Since we can freely control the conversion of the post-polycondensation and SCAT reaction, we can optimize the conditions and obtain the best structure. In addition to the control of the nanospaces, we can select the base polymers. Therefore, using these methods, we can obtain the best performance not only for oxygen permselective membranes, but also for other gas permselective membranes based on the molecular sieving mechanism.

## Figures and Tables

**Table 1:** Characterization and oxygen permselectivity of the composite membranes of novel nanoporous polycondensates (sc(**I/Si**)) based on PDPA prepared according to method 1 (HSP method) (Scheme 5).

No.	Additives	Content (wt%)	*θ*^a)^ (deg)	*P*_O2_^b)^ (barrer)	*α* ^b)^
1	none(pure PDPA)	0	103	1520	1.83
2	scat(**I**/**Si**)	1.0	91.9	3120	2.15
3	sc(**I**/**Si**)	1.0	90.5	5320	2.45
4	none(pure scat(**I**/**Si**))	0	90.1	- ^c)^	- ^c)^

^a)^ Contact angles for water droplets on the blend membranes. ^b)^ 1 barrer = 10^−10^ cm^3^ (STP) cm cm^−2^ s^−1^ cmHg^−1^, *α* = *P*_O2_/*P*_N2_. ^c)^ No self-standing membrane-forming ability.

**Table 2:** Characterization and oxygen permselectivity of the composite membranes of novel nanoporous polycondensates (sc(**V’**/**EO**)) based on PVAc prepared according to method 1(HSP method) (Scheme 5).

No.	Additives	*M*_w_ of the Additives ^a)^	Content (wt%)	*P*_O2_^b)^ (barrer)	*α* ^b)^
1	none (pure PVAc)	none	0	5.58	2.97
2	scat(**V**’/**EO**)	1710	10	6.31	3.99
3	sc(**V**’/**EO**)	3490	10	6.52	4.12

^a)^ By GPC(THF, PSt standard), see Figure S1. ^b)^ 1 barrer = 10^−10^ cm^3^ (STP) cm cm^−2^ s^−1^ cmHg^−1^, *α* = *P*_O2_/*P*_N2_.

**Table 3:** Characterization of novel nanoporous polycondensates (SiO-sc(**V**)) and oxygen permselectivity of the composite membranes of SiO-sc(**V**) based on PDPA prepared according to method 2 (HPS method) (Scheme 6).

SiO-sc(V)				Composite Membrane			
No.	*M*_w_ of the Peak Having the Highest *M*_w_^a)^ (×10^5^)	Contents of SiO-sc(V) ^a)^ (%)	Composition of sc(V) in SiO-sc(V) ^b)^ (%)	Content of SiO-sc(V) (wt%)	*θ*^c)^ (deg)	*P*_O2_^d)^ (barrer)	*α* ^d)^
1	5.74	71.1	39.9	0.5	96.6	1770	2.49
2	5.74	71.1	39.9	1.0	96.5	1920	2.28
3 ^e)^	-	-	-	0	103	1520	1.83
4 ^f)^	0.08	-	-	100	86.2	- ^g)^	- ^g)^

^a)^ Determined by GPC(THF, PSt standard), see Figure 1. ^b)^ By IR. ^c)^ Advancing contact angles for water droplets on the air surface of the blend membranes. ^d)^ 1 barrer = 10^−10^ cm^3^ (STP) cm cm^−2^ s^−1^ cmHg^−1^, *α = P*_O2_/*P*_N2_. ^e)^ Pure PDPA membrane. ^f)^ Pure sc(**V**) membrane, see Table 4. ^g)^ No self-standing membrane-forming ability.

**Table 4:** Characterization of low molecular weight polycondensates (sc(**V**)) and oxygen permselectivity of the composite membranes of the sc(**V**) having no large nanopores based on PDPA prepared according to method 3 (Scheme S1).

sc(V)				Composite Membrane			
No.	Additives	Peak for Higher *M*_w_^a)^ (×10^3^)	Area Ratio of *M*_w max_^a)^ (%)	Content ^b)^ (wt%)	*θ*^c)^ (deg)	*P*_O2_^d)^ (barrer)	*α* ^d)^
1	sc(**V**)	8.18	75.2	1.0	98.4	1250	2.16
2	scat(**V**)	1.30	-	1.0	97.3	1190	2.14

^a)^ By GPC (THF, PSt standard). ^b)^ the content of the additives. ^c)^ Advancing contact angles for water droplets of the composite membranes. ^d)^ 1 barrer = 10^−10^ cm^3^ (STP) cm cm^−2^ s^−1^ cmHg^−1^, *α = P*_O2_/*P*_N2_.

**Table 5:** Characterization of sc(**I/Si**), SiO-sc(**V**), and sc(**V**) and oxygen permselectivity (*α*) of their PDPA-based composite membranes prepared by the three methods, respectively.

sc(R_f_)					Composite Membrane	
Method	Code for sc(R_f_)	Size of the Nonopores or Nanospaces ^a)^ (nm)	Peak for Higher *M*_w_^b)^ (×10^3^)	Area Ratio of *M*_w max_^b)^ (%)	*θ*^c)^ (deg)	*α* ^d)^
1	sc(**I**/**Si**)	3.8~4.6	- ^e)^	- ^e)^	90.5 (90.1)	2.45
2	SiO-sc(**V**)	2.5~3.3	574	71.1	96.6 (86.2)	2.49
3	sc(**V**)	0.31~0.36	8.18	75.2	98.4 (86.2)	2.16

^a)^ See Figure 2 and Figure 6, Figure S2. ^b)^ By GPC(THF, PSt standard). ^c)^ Advancing contact angles for water droplets on the PDMS-based composite membranes. The values in the parentheses are for pure scat(**I**/**Si**) or scat(**V**) membranes. ^d)^ *α* = *P*_O2_/*P*_N2_. ^e)^ no data because separation of sc(**I**/**Si**) and PDPA was impossible.

## Supplementary Materials

The Supplementary Materials are available online at https://www.mdpi.com/2079-4991/9/6/859/s1, Scheme S1, Figures S1–S3 and the experimental procedure for preparation of composite membranes of low molecular weight sc(**V**) (oligocondensates) having no large nanopores (method 3; Scheme S1).
