# Supplementary material for: New Synthetic Methods of Novel Nanoporous Polycondensates and Excellent Oxygen Permselectivity of Their Composite Membranes
Source: Nanomaterials (Basel). 2019 Jun 5;9(6):859. doi: 10.3390/nano9060859 (PMC6631047; doi:10.3390/nano9060859)
Supplement: Supplementary file 1 [file nanomaterials-09-00859-s001.pdf]

Supplementary materials

New Synthetic Methods of Novel Nanoporous Polycondensates and Excellent Oxygen Permselectivity of Their Composite Membranes

Yu Zang [1,*], Toshiki Aoki [2,*], Masahiro Teraguchi [2], Takashi Kaneko [2], Hongge Jia [1], Liqun Ma [1] and Fengjuan Miao [3]

[1] Heilongjiang Province Key Laboratory of Polymeric Composition Material, College of Materials Science and Engineering, Qiqihar University, Wenhua Street 42, Qiqihar 161006, China; jiahongge11@hotmail.com (H.J.); maliqun6166@163.com (L.M.)

[2] Faculty of Engineering, Niigata University, Ikarashi 2-8050, Nishi-ku, Niigata 950-2181, Japan; teraguti@eng.niigata-u.ac.jp (M.T.); kanetaka@gs.niigata-u.ac.jp (T.K.)

[3] College of Communications and Electronics Engineering, Qiqihar University, Wenhua Street 42, Qiqihar 161006, China; miaofengjuan@163.com

* Correspondence: zangyu@qqhru.edu.cn (Y.Z.); toshaoki@eng.niigata-u.ac.jp (T.A.)

Contents

1. Supplementary scheme and figures

2. Experimental procedure for preparation of composite membranes of low molecular weight sc(**V**)(oligocondensates) having no large nanopores (method 3; Scheme S1).

   2.1 Synthesis of low molecular weight sc(**V**) having no large nanopores by *in situ* ADMET of scat(**V**) in the membrane state

   2.2 Preparation of composite membranes consisting of low molecular weight sc(**V**) having no large nanopores and PDPA

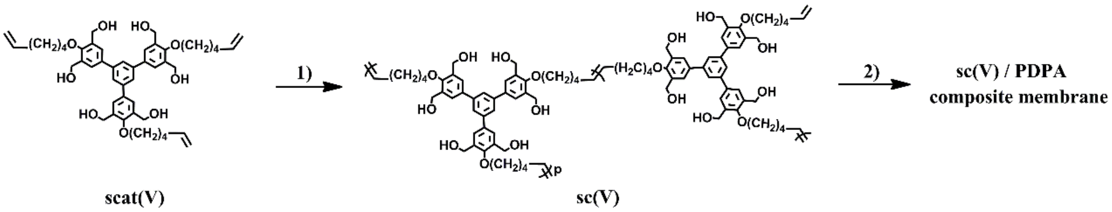

Scheme S1. Preparation route to composite membranes of oligocondensates having no large nanopores (method 3): 1) oligocondensation of pure scat(**V**) membrane, which had been prepared by irradiation of poly(**V**)(2.1.1), with the Grubbs catalyst without any template to yield low molecular weight soluble sc(**V**), 2) Fabrication of the composite membranes of soluble sc(**V**) with a base polymer (PDPA).

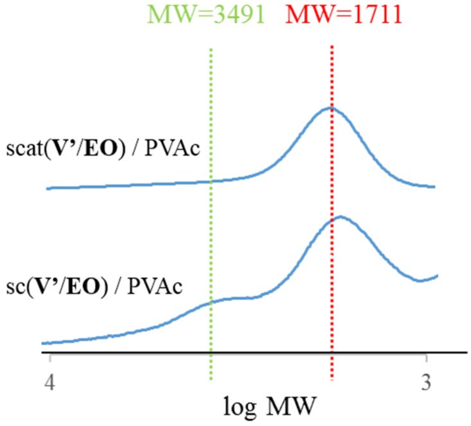

Figure S1. GPC charts (detected by UV) of the blend solution of sc(**V′/EO**) or scat(**V′/EO**) and PVAc.

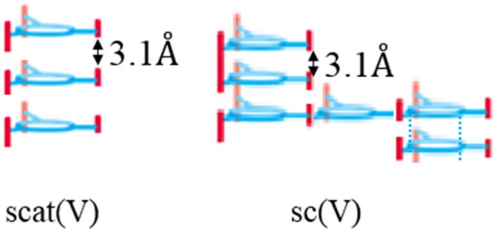

Figure S2. Possible small nanospace in sc(**V**) by oligocondensation of scat(**V**) with no templates in its pure membrane states.

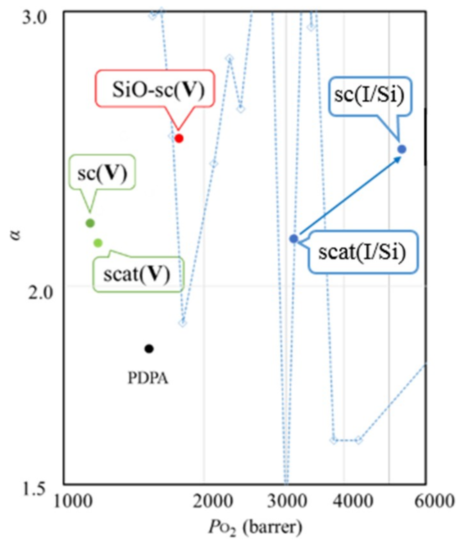

Figure S3. Oxygen permeation behavior through the composite PDPA based membranes of sc(**I/Si**)(method 1), SiO-sc(**V**) (method 2), and low molecular weight sc(**V**) (method 3) prepared by the three methods. ------ : upper boundary plots from the literature (2016)

2. Experimental procedure for preparation of composite membranes of low molecular weight sc(V)(oligocondensates) having no large nanopores (method 3; Scheme S1)

2.1 Synthesis of low molecular weight sc(V) having no large nanopores by in situ ADMET of scat(V) in the membrane state

2.1.1 Synthesis of scat(**V**) by SCAT

A poly(**V**) membrane was irradiated under nitrogen by visible light ((400-500 nm, 23000 lx) for 7 days. The completion of the SCAT reaction was monitored by GPC detected by UV. The resulting poly(**V**) membrane was used for ADMET reaction(2.1.1) as it was. GPC: M_n=1.57x10^3; M_w=1.70x10^3; M_w/M_n=1.09. IR(cm^{-1}, film): 3600-3100(-OH), 3078(-CH=CH_2), 2925, 2854(CH_2), 1641(C=C), 1464(C-O-C). ^{1}H NMR (700 MHz, $CDCl_3$, ppm) δ: 1.63(br, 5.59H, -C<u>H</u>$_2$CH$_2$CH=CH$_2$), 1.86(quint, 6.74H, -C<u>H</u>$_2$CH$_2$CH$_2$CH=CH$_2$), 2.17(q, 7.17H, -C<u>H</u>$_2$CH=CH$_2$), 3.96(br, 6.59H, -OC<u>H</u>$_2$CH$_2$CH$_2$CH$_2$CH=CH$_2$), 4.43(br, 11.5H, Ph-C<u>H</u>$_2$-OH), 5.00-5.09(dd, 6.22H, -CH=C<u>H</u>$_2$), 5.86(tdd, 3.46H, -C<u>H</u>=CH$_2$), 6.75(br, 6.23H, Ph<u>H</u>-O(CH$_2$)$_4$), 6.86(br, 3.00H, Ph<u>H</u> of trisubstituted benzene).

2.1.2 Synthesis of sc(**V**) by ADMET of scat(**V**) membrane (Scheme S1, 1))

The membrane of scat(**V**) (21.1 mg), which had been prepared by irradiation of poly(**V**)(2.1.1), was immersed into toluene (10.0 mL) solution of Hoveyda-Grubbs catalyst

2st generation (6.27mg) (1.00 mM) for 24h. After the reaction, the membrane was washed with toluene, and then immersed into methanol. The soluble part in methanol was removed and then the insoluble membrane was immersed into THF. This time, the insoluble part in THF was removed, the filtrate was evaporated and dried in *vacuo* for 24 h to give sc(**V**) with yield of 3.8%. GPC: peak for higher M_n and M_w, M_n=3.20×10^3, M_w=8.18×10^3, M_w / M_n =2.56, area ratio = 75.2%.

2.2 Preparation of composite membranes consisting of low molecular weight sc(V) having no large nanopores and PDPA (Scheme S1, 2))

A toluene solution (3.00 mL) of the base polymer PDPA (49.5 mg) and THF solution (0.100 mL) of the sc(**V**) (0.500 mg, 1.0 wt%) were blended together. And then the mixed solution was cast on a poly(tetrafluoroethylene) sheet (100 cm^2). After the solvent was evaporated for 24 h at room temperature, the membrane was detached from the sheet and dried in *vacuo* for 24 h at room temperature.
